# Knockout of RNA Binding Protein MSI2 Impairs Follicle Development in the Mouse Ovary: Characterization of MSI1 and MSI2 during Folliculogenesis

**DOI:** 10.3390/biom5031228

**Published:** 2015-06-26

**Authors:** Jessie M. Sutherland, Alexander P. Sobinoff, Kara M. Gunter, Barbara A. Fraser, Victoria Pye, Ilana R. Bernstein, Evan Boon, Nicole A. Siddall, Luisa I. De Andres, Gary R. Hime, Janet E. Holt, Thomas Graf, Eileen A. McLaughlin

**Affiliations:** 1 School of Environmental and Life Sciences, University of Newcastle, Callaghan NSW 2308, Australia; E-Mails: jessie.sutherland@newcastle.edu.au (J.M.S.); asobinoff@cmri.org.au (A.P.S.); Kara.gunter@uon.edu.au (K.M.G.); barbara.fraser@newcastle.edu.au (B.A.F.); victoria.pye@newcastle.edu.au (V.P.); ilana.bernstein@newcastle.edu.au (I.R.B.); 2 School of Biomedical Sciences & Pharmacy, University of Newcastle, Callaghan NSW 2308, Australia; E-Mails: evan.boon@uon.edu.au (E.B.); janet.holt@newcastle.edu.au (J.E.H.); 3 Anatomy and Neuroscience, University of Melbourne, Parkville VIC 3010, Australia; E-Mails: n.siddall@unimelb.edu.au (N.A.S.); g.hime@unimelb.edu.au (G.R.H.); 4 Differentiation and Cancer Program, Center for Genomic Regulation and UPF, Barcelona 08000, Spain; E-Mails: marisa.deandres@crg.eu (L.I.D.A.); Thomas.Graf@crg.eu (T.G.)

**Keywords:** fertility, Musashi, oocyte, granulosa cell, oogenesis

## Abstract

Characterizing the mechanisms underlying follicle development in the ovary is crucial to understanding female fertility and is an area of increasing research interest. The RNA binding protein Musashi is essential for post-transcriptional regulation of oocyte maturation in *Xenopus* and is expressed during ovarian development in *Drosophila*. In mammals Musashi is important for spermatogenesis and male fertility, but its role in the ovary has yet to be characterized. In this study we determined the expression of mammalian Musashi proteins MSI1 and MSI2 during mouse folliculogenesis, and through the use of a MSI2-specific knockout mouse model we identified that MSI2 is essential for normal follicle development. Time-course characterization of MSI1 and MSI2 revealed distinct differences in steady-state mRNA levels and protein expression/localization at important developmental time-points during folliculogenesis. Using a gene-trap mouse model that inactivates *Msi2*, we observed a significant decrease in ovarian mass, and change in follicle-stage composition due to developmental blocking of antral stage follicles and pre-antral follicle loss through atresia. We also confirmed that hormonally stimulated *Msi2*-deficient mice produce significantly fewer MII oocytes (60.9% less than controls, *p* < 0.05). Furthermore, the majority of these oocytes are of poor viability (62.2% non-viable/apoptotic, *p* < 0.05), which causes a reduction in female fertility evidenced by decreased litter size in *Msi2*-deficient animals (33.1% reduction to controls, *p* < 0.05). Our findings indicate that MSI1 and MSI2 display distinct expression profiles during mammalian folliculogenesis and that MSI2 is required for pre-antral follicle development.

## 1. Introduction

The choice to postpone pregnancy until later in life is a growing trend observed among women in developed countries, which is contributing to an increasing incidence of couples seeking fertility treatment [1]. Women reach a peak in fertility during their early twenties, followed by a gradual decline in fertility to the approximate age of 35 [2]. Beyond this point infertility increases until menopause is reached at around 50 years of age [3]. Thus understanding the mechanisms underlying female fertility is an area of increasing research interest.

The female reproductive lifespan is limited by the finite number of primordial follicles that form within the ovary during late-gestational development in humans, or shortly after birth in the mouse [4]. These follicles undergo continuous programmed cell death, termed “atresia”, with less than 0.1% of the follicles present at birth reaching the ovulatory stage [5]. Follicle atresia is commonly initiated within the somatic granulosa cell layers that surround the growing oocyte [6]. Granulosa cells provide a supportive environment for the oocyte, responsible for the production of sex steroids and growth factors necessary for development, with widespread granulosa cell loss resulting in follicle death. The regulatory factors controlling granulosa-mediated atresia remain unknown.

The Musashi family of RNA binding proteins plays important roles in stem cell function and cell fate determination in mammalian systems [7,8,9,10,11], and is considered to be fundamental to the regulation of oocyte maturation in *Xenopus* [12,13,14,15]. However, despite assessment during male reproductive development [16,17] and confirmed ovarian expression in *Drosophila* [18], a potential role for Musashi during mammalian folliculogenesis has yet to be considered. The aim of this study was to: (1) characterize the expression of Musashi proteins MSI1 and MSI2 during folliculogenesis in the mouse, serving as a model for mammalian development and (2) determine if MSI2 is essential for normal follicle development, utilizing an *Msi2* gene-trap mouse model.

## 2. Results and Discussion

### 2.1. MSI1 and MSI2 Time-Course Characterization

**Figure 1:**
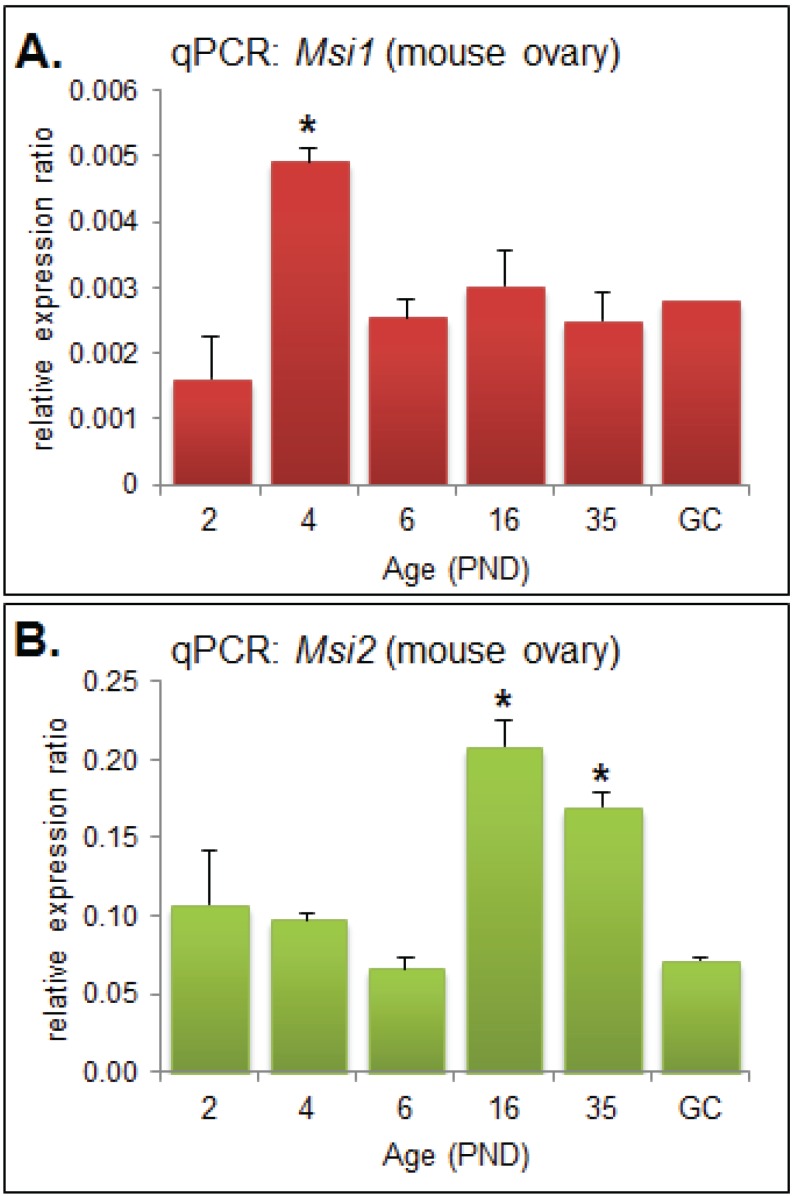
Quantitative PCR (qPCR) Analysis of Musashi during ovary maturation*. (***A**) QPCR of *Msi1* in ovary time-course from post-natal day (PND) 2 through to sexual maturity at PND35 and in isolated granulosa cells (GC). MRNA expression relative to *Cyclophilin A* (2eΔCt); values are mean + SEM; (ovary: *n* = 3, GC: *n* = 2). (**B**) QPCR of *Msi2* in ovary time-course from PND 2 through to PND35 and in isolated granulosa cells (GC). Fold change relative to *Cyclophilin A* (2eΔCt); values are mean + SEM; (ovary: *n* = 3, GC: *n* = 2).

Quantitative PCR (qPCR) was performed on RNA extracted from mouse ovaries and oocytes collected at developmentally important post-natal day (PND) time points [19] in order to observe the steady-state mRNA expression of *Msi1* and *Msi2*. *Msi1* steady-state mRNA was most highly expressed at PND4 (*p* < 0.05), and otherwise detected at relatively low levels for all other time points (Figure 1A). At PND4 the ovary is composed primarily of primordial follicles, with some follicles beginning to activate and transition to primary stage. *Msi2* steady-state mRNA expression was significantly enriched at PND16 and PND35 (*p* < 0.05) relative to the earlier time-points (Figure 1B). PND16 and PND35 mark the transition to sexual maturation; the ovary consists of a higher proportion of growing follicles at advanced pre-antral or antral stage, respectively, and is comprised of an enriched population of granulosa and stromal cells.

**Figure 2:**
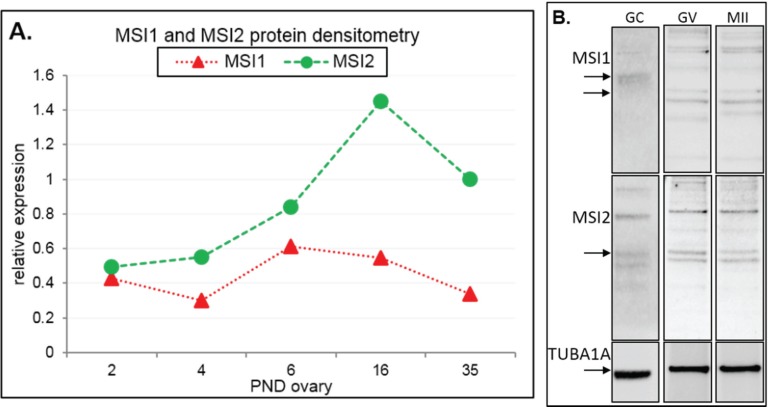
Immunoblot analysis of Musashi during ovary maturation. (**A**) Protein densitometry of full-length MSI1 and MSI2 in ovary time-course from post-natal day (PND) 2 through to sexual maturity at PND35. Protein expression relative to TUBA1A. For original immunoblots see Supplemental Figure S2. *n* = 1. (**B**) Immunoblot of MSI1 and MSI2 in isolated granulosa cells (GC), germinal vesicle oocytes (GV), and *in vitro* matured metaphase II oocytes (MII). Positive expression of MSI1 was detected at the appropriate size of 39 kDa for isoform 1 and 34 kDa for isoform 2 (only in oocytes). Positive expression of MSI2 was detected at ~37 kDa. Protein expression was measured relative to TUBA1A loading control at 55 kDa. For immunoblots with molecular weight marker, see Supplemental Figure S2.

**Figure 3:**
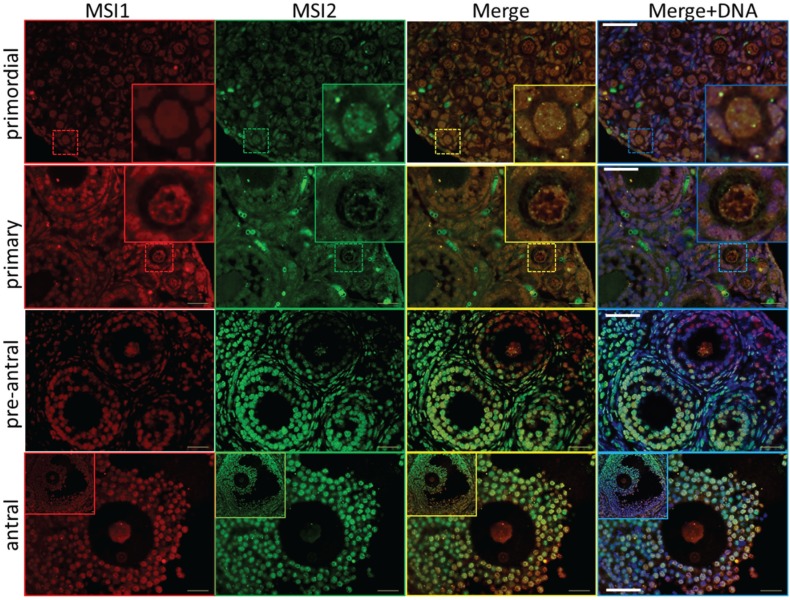
Immunolocalization of Musashi during ovary maturation. Mouse ovary sections were probed for antibodies against MSI1 (red) and MSI2 (green), and counter-stained with DNA marker DAPI (blue). Based on ovary age and follicle composition, images were classified developmentally into primordial, primary, pre-antral, and antral. Sections were visualized via epifluorescent microscopy. Insets in primordial and primary sets show zoomed image of follicle-type. Scale bar in merged images = 40 μm.

**Figure 4:**
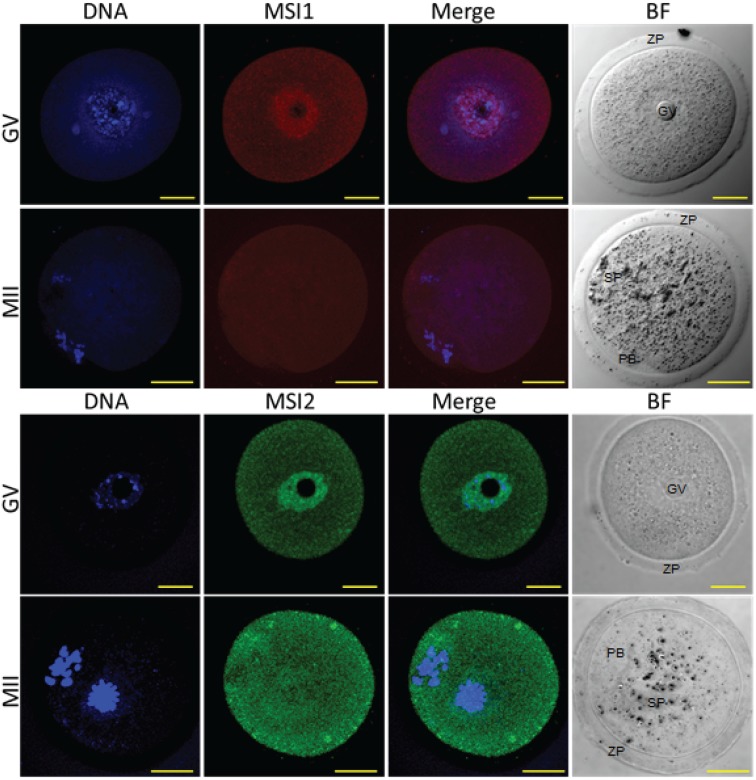
Immunocytochemistry of Musashi in germinal vesicle (**GV**) and metaphase II (**MII**) oocytes. Mouse oocytes were probed for antibodies against MSI1 (red) and MSI2 (green), and counter-stained with DNA marker Hoechst (blue). Representative images show Z-stack of total oocyte. Bright-field (BF) images were taken alongside confocal immunofluorescent microscopy. In BF images GV = germinal vesicle, ZP = zona pellucida, PB = polar body, SP = MII spindle. Scale bar = 20 μm. Consistent for *n* = 10.

Steady-state protein expression and localization of MSI1 and MSI2 in mouse ovaries and oocytes were measured via immunoblot and immunofluorescence. Densitometry to compare protein expression across a range of ovarian time-points was quantified relative to the housekeeping protein TUB1A1. MSI1 analysis indicated that expression was enriched at PND6, with lower relative expression detected in mature ovaries at PND35 (Figure 2A and Supplementary Figure S2). MSI2 analysis indicated that expression increased as the ovary developed towards adult maturation, with relatively low levels of MSI2 detected in the early PND ovary (Figure 2A and Supplementary Figure S2). Expression of MSI1 and MSI2 was also detected in isolated granulosa cells and both immature GV (germinal vesicle) stage and mature metaphase II (MII) stage oocytes (Figure 2B). Interestingly, the MSI1 product observed in isolated oocytes was detected at a slightly smaller molecular weight than the 39 kDa full-length product observed in total ovary protein (Supplementary Figure S2) and isolated granulosa cells. Immunofluorescence of MSI1 in ovarian tissue showed localization primarily to the nucleus of granulosa cells and oocytes from primordial to pre-ovulatory follicles with decreasing intensity (Figure 3). In isolated oocytes MSI1 was detected throughout the cytoplasm, and intensely in the nucleus of GV oocytes (Figure 4); it was expressed faintly throughout cytoplasm of MII oocytes (Figure 4). In ovarian tissue MSI2 localized mostly to the nucleus of granulosa cells and oocytes from primordial to pre-ovulatory follicles, but with increasing intensity, with expression observed strongly in the granulosa cells of antral stage follicles (Figure 3). Similar to MSI1, MSI2 was present in the cytoplasm of GV oocytes, with increased intensity in the nucleus. MSI2 was also observed throughout the MII oocyte cytoplasm, with the notable presence of intensely stained cytoplasmic aggregates (Figure 4).

### 2.2. Analysis of Folliculogenesis in an Msi2-Deficient Mouse Model

Msi2-deficient mice were gifted by Professor Thomas Graf, group leader of the Center for Genomic Regulation and UPF, Barcelona, Spain. Generated via retroviral gene-trap insertion, *Msi2*-deficient mice (Msi2^Gt/Gt^) evidenced 50% embryonic lethality, 20% reduced body weight, and sub-fertility [20]. Depletion of *Msi2* gene expression in the ovary of Msi2^Gt/Gt^ mice was verified by qPCR (Supplementary Figure S3A). Complete loss of full-length MSI2 protein expression in the ovary of Msi2^Gt/Gt^ mice was confirmed by immunoblot (Supplemental Figure S3B).

**Figure 5:**
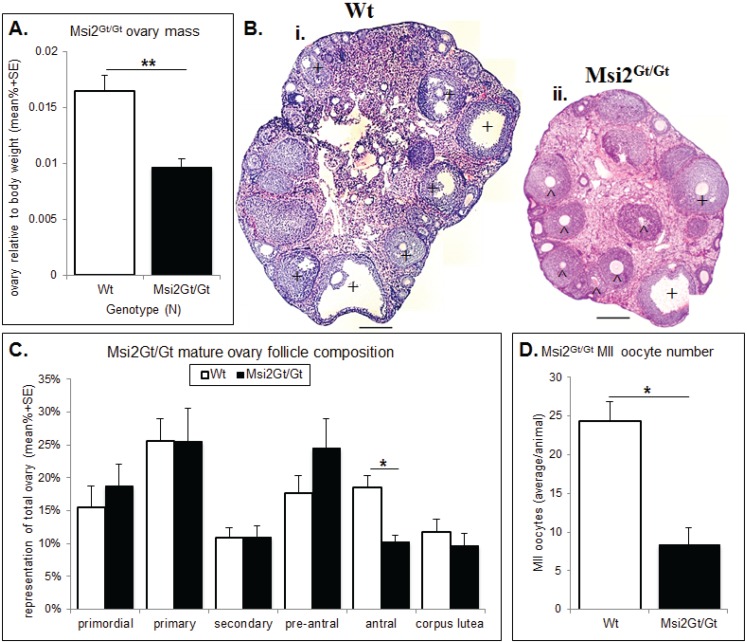
Histological analysis of the Msi2^Gt/Gt^ ovary and oocytes. (**A**) Mature Msi2^Gt/Gt^ (*n* = 5) and Wt (*n* = 8) ovary mass relative to body weight. Value presented as mean relative ovary mass/female/genotype + SE, **: denotes *p* < 0.01. (**B**) H & E image of Wt (**i**) and Msi2^Gt/Gt^ (**ii**) adult ovary cross-sections. Scale bar = 200 μm. ^ indicates increased # of pre-antral follicles + indicates decreased # of antral follicles in Msi2^Gt/Gt^ ovary relative to Wt. (**C**) Mature Msi2^Gt/Gt^ and Wt follicle counts, values presented as mean % composition + SE, *: denotes *p* < 0.05). *n* = 7. (**D**) Msi2^Gt/Gt^ (*n* = 9) and Wt (*n* = 7) MII oocyte collection counts. MII oocytes were collected from the oviductal ampullae of super-ovulated adult mice and immediately counted, values are average oocytes/animal + SE, *: denotes *p* < 0.05.

Histological analysis of the Msi2^Gt/Gt^ ovary revealed on average a 41% reduction in total ovarian mass relative to body weight compared to wild-type (Wt) littermates (*p* < 0.01) (Figure 5A). This was concomitant with a 36.0% decrease in total follicle number in mature Msi2^Gt/Gt^ ovaries relative to Wt littermates (*p* < 0.05) (Supplementary Figure S4). Follicle counts indicated an over-representation of pre-antral follicles and a significant reduction in antral/pre-ovulatory follicles in Msi2^Gt/Gt^ mature ovaries (*p* < 0.05) (Figure 5C). Examination of H & E stained sections verified an increase in the proportion of pre-antral follicles of unhealthy appearance observed in the Msi2^Gt/Gt^ mature ovary (Figure 5Bi & ii). The average number of MII oocytes collected from Msi2^Gt/Gt^ super-ovulated mice was also significantly reduced relative to Wt littermates (*p* < 0.05) (Figure 5D).

**Figure 6:**
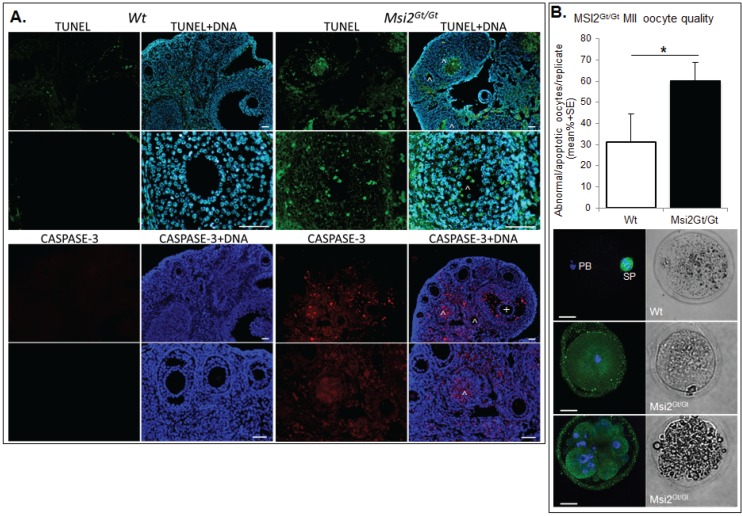
Assessment of Msi2^Gt/Gt^ ovarian and oocyte health. (**A**) Immunofluorescence of TUNEL and CASPASE-3 in Msi2^Gt/Gt^ and Wt adult ovary. Representative images demonstrate an increase in TUNEL (green) and CASPASE-3 (red) in Msi2^Gt/Gt^ pre-antral (^) and antral (^+^) follicles, verified via quantitative analysis *n* = 4. Scale bar = 50 μm; (**B**) Msi2^Gt/Gt^ and Wt MII oocyte quality. MII oocytes collected from the oviductal ampullae of super-ovulated adult mice were fixed and subjected to immunocytochemistry for α-tubulin (green) to identify spindles and Hoechst (blue) to identify polar body DNA and oocyte chromosomes. Representative images show typical MII staining pattern in Wt depicting polar body (PB) formation and normal chromosome and spindle alignment (SP). Msi2^Gt/Gt^ images depict irregular spindle assembly and chromosome alignment and oocyte apoptosis. *n* = 3 biological replicates, 3 mice/replicate, *: denotes *p* < 0.05. Scale bar = 20 μm.

Ovarian and oocyte health was quantitated using established markers of DNA damage, apoptosis, and MII oocyte quality. Immunofluorescence of TUNEL positive follicles indicated a significant 24.2% increase in follicles exhibiting DNA damage within Msi2^Gt/Gt^ adult ovarian sections when compared to Wt controls (*p* ≤ 0.05) (Figure 6A). This correlated with a 28.3% enrichment of activated CASPASE 3 positive immunofluorescence in Msi2^Gt/Gt^ follicles, indicating increased cellular apoptosis (*p* = 0.06) (Figure 6A). MII oocyte quality was analyzed via spindle characterization and DNA immunofluorescence. Msi2^Gt/Gt^ oocytes displayed a 29.0% increase in the average number of apoptotic/abnormal oocytes compared to Wt littermates (*p* = 0.11) (Figure 6B).

**Figure 7:**
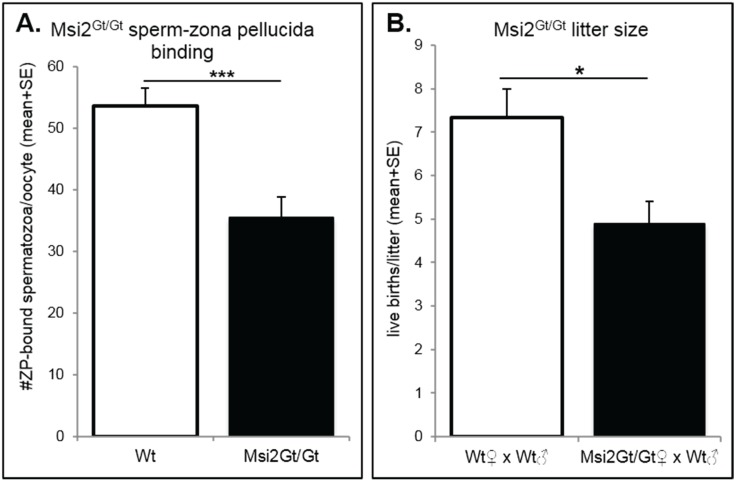
Assessment of Msi2^Gt/Gt^ fertility. (**A**) Sperm-zona pellucida binding in Msi2^Gt/Gt^ (*n* = 8) and Wt (*n* = 9) MII oocytes. MII oocytes were collected from the oviductal ampullae of super-ovulated adult mice and subjected to sperm zona-binding with capacitated mature epididymal spermatozoa from control adult males. Following incubation and washing, the number of sperm that remained bound to the zona pellucida was counted. ***: denotes *p* < 0.001. (**B**) Average litter size in Msi2^Gt/Gt^ and Wt females. Breeding pairs were assembled, following genotyping, at ~5 weeks and pairs were kept together for up to 6 months. The average number of live births per litter was recorded. *n* = 3 individual Wt × Wt breeding pairs, *n* = 10 individual Msi2^Gt/Gt^ (female) and Wt (male) pairs. * denotes *p* < 0.05.

The fertility of Msi2^Gt/Gt^ mice was assessed using sperm-zona pellucida (ZP) binding assays and through randomized breeding trials. Msi2^Gt/Gt^ oocytes demonstrated a significant 33.9% decrease in the binding of control spermatozoa to the MII oocyte ZP *in vitro* compared to Wt oocytes (*p* ≤ 0.001), representing a reduction in fertilization potential (Figure 7A). Adult Msi2^Gt/Gt^ females exhibited a 33.2% decrease in average litter size generated from randomized breeding trials with Wt males (Figure 7B). This was a significant reduction from control Wt/Wt crosses (*p* < 0.05).

### 2.3. Discussion

This study has successfully characterized the expression of the Musashi RNA binding proteins during folliculogenesis. Furthermore, through the use of a *Msi2* knockout mouse [20], we have shown extensive evidence for the requirement of MSI2 for normal follicle development. We have confirmed that MSI1 and MSI2 are expressed throughout the developing mouse ovary in both granulosa cells and oocytes at the mRNA and protein level. The expression of MSI1 and MSI2 during folliculogenesis appears to be differential, with MSI1 expression more abundant during early post-natal development in primordial and primary follicles, while MSI2 was detected at much higher levels in pre-antral and antral follicles. MSI1 and MSI2 were both expressed at the GV stage in isolated oocytes. MSI2 continued to be highly expressed in MII oocytes, while MSI1 expression was detected at low levels. Assessment of our Msi2^Gt/Gt^ mouse model indicated that loss of full-length MSI2 protein production has a profound impact on folliculogenesis. Msi2^Gt/Gt^ adult mice had smaller ovaries with decreased total follicle population and MII oocyte number. A lower overall percentage of antral-stage follicles was observed, indicating that folliculogenesis is potentially blocked at pre-antral stage. The antral follicles that do develop appear to be of poor health, as confirmed by the increase in DNA damage and apoptosis in Msi2^Gt/Gt^ ovaries and oocytes. The reduced number and quality of oocytes produced by Msi2^Gt/Gt^ mice negatively impacted on fertility through decreased spermatozoa-zona pellucida binding and reduced litter sizes.

Our robust experimental design, utilizing multiple techniques for the analysis of expression and assessment of folliculogenesis, establishes MSI2 as a key player in normal ovarian function. In our *Msi2*-deficient mouse model we were limited by our inability to attribute our observed phenotype to the loss of MSI2 in oocytes, granulosa cells, or both. Our findings have indicated that MSI2 is highly expressed in both cells types in growing pre-antral and antral follicles and when MSI2 is absent we see a decline in ovarian health, predominately through apoptosis of these follicle stages. The granulosa cells of these follicles displayed the greatest damage and we suggest that the observed poor quality oocyte production is attributable to the inadequate environment provided by surrounding MSI2-depleted granulosa cells. It is well established that the quality of follicular oocytes is dependent on interactions with surrounding somatic granulosa cells. Granulosa cells form gap junction projections, allowing transport of metabolites to and from the oocyte [21], and are responsible for the steroidogenic activity of the maturing follicle [22], expressing receptors necessary for triggering final follicular maturation and ovulation [23]. Furthermore, the expression levels of some genes in granulosa cells are predictive of oocyte developmental potential [24,25]. Future research into the role of MSI2 in folliculogenesis would benefit from the study of granulosa cell and oocyte cell specific knockouts [26,27].

Previous studies have highlighted the importance of RNA binding proteins in gametogenesis [28,29,30], with established roles for Musashi in *Xenopus* oocyte development [15] and mammalian spermatogenesis [16]. Oocytes are transcriptionally inactive at the mature GV stage and during meiotic maturation to MII stage [31]. Consequently, post-transcriptional regulation, controlled by specifically expressed RNA binding proteins, is essential for normal development during oocyte maturation [28]. RBPs with established roles in controlling and coordinating follicular development, oocyte growth, and meiotic maturation include the OMA proteins in *C. elegans* [32,33], the germ cell-specific DAZL proteins [30,34], SAM68 [35], and the PABP family [30,36]. Like Musashi, the majority of these RBPs are highly conserved across species, demonstrating the prevalence of RBPs in post-transcriptional mechanisms to effect changes in gene expression. In immature *Xenopus* oocytes, expression of a dominant inhibitory form of Musashi abolished meiotic cell cycle progression and prevented germinal vesicle breakdown [15]. Our studies similarly demonstrated that the deletion of *Msi2* in our Msi2^Gt/Gt^ mouse model resulted in reduced mature follicle development and decreased production of mature MII oocytes. Our previous studies detailing the expression of MSI1 and MSI2 throughout spermatogenesis revealed that both proteins occupied different functional niches, while germ cell-specific overexpression of *Msi2* significantly increased DNA damage and apoptosis, causing sterility [16]. In this study we have likewise established the differential expression of MSI1 and MSI2 throughout folliculogenesis, and similarly demonstrated through loss of *Msi2* the negative impact on follicle health and development and fertility. These combined findings establish different roles for MSI1 and MSI2 in reproductive tissues and support an important function for MSI2 in mammalian gametogenesis.

Our mouse models provide a mammalian example of the importance of Musashi RBPs in regulating follicle development and controlling fertility. The mouse ovary and the hormonal regulation of folliculogenesis and oocyte maturation are comparable to humans in many key aspects. Furthermore, emerging evidence suggests that translational regulation plays a similarly important role in human germ cells and fertility [30,37].

This study has determined the presence and differential expression of MSI1 and MSI2 in the developing ovary and oocyte and established that MSI2 is required for healthy follicle and oocyte development and fertility. Future research should focus on the mRNA targets of MSI1 and MSI2 during folliculogenesis and elaborate on the regulatory mechanisms utilized by both proteins.

## 3. Experimental Section

*Animals*: Use of animals for this study was approved by the University of Newcastle Animal Care and Ethics Committee. All animals were maintained according to the recommendations prescribed by the ACEC. Prior to dissection, animals were euthanized via CO_2_ asphyxiation in accordance with ACEC directives.

*Chemicals and Reagents*: All chemicals, media, and reagents were obtained from Sigma (Sigma-Aldrich, St. Louis, MO, USA), Promega (Promega, Madison, WI, USA), and Life Technologies (Thermo Fisher Scientific Corporation, Carlsbad, CA, USA) unless otherwise stated.

*RNA Extraction, Reverse Transcription, Quantitative PCR*: Total RNA isolation was performed using two rounds of a modified acid guanidinium thiocyanate-phenol-chloroform protocol followed by isopropanol precipitation [38]. Reverse transcription was performed as previously described [39]. Total RNA was treated with DNase prior to reverse transcription to remove genomic DNA. Reverse transcription reactions were verified by ß*-actin* RT-PCR using cDNA amplified with GoTaq Flexi. QPCR was performed using SYBR Green GoTaq qPCR master mix according to manufacturer’s instructions on LightCycler 96 SW 1.0 (Roche, BASEL, Switzerland). Primer sequences have been supplied (see Supplementary Table S1). Reactions were performed on cDNA equivalent to 50 ng of total RNA and carried out for 45 amplification cycles. SYBR^®^ Green fluorescence was measured after the extension step at the end of each amplification cycle and quantified using LightCycler Analysis Software (Roche). For each sample, a replicate omitting the reverse transcription step was undertaken as a negative control. QPCR data was normalized to the house-keeping control *Cyclophilin A.* Experiments were replicated at least 3 times prior to statistical assessment. Each PCR was performed on at least 3 separate cell or tissue isolations, of which a representative PCR or an average is shown.

*Protein Extraction, Immunoblot, Protein Densitometry:* Protein was extracted using an RIPA lysis buffer (150 mM sodium chloride, 0.5% sodium deoxycholate, 1.0% Triton-X, 0.1% SDS, 50 mM Tris, pH 8.0, Protocease protease inhibitor (G-Biosciences St. Louis, MO, USA)). Protein concentration was estimated using a Pierce BCA Protein Assay Kit [40]. Immunodetection was conducted as previously described [41], using primary antibodies for MSI1 (AF2628, R&D systems, Minneapolis, MN, USA), pan-MSI2 (ab50829, Abcam), or full-length MSI2 (ab76148, Abcam, Cambridge, UK) (used in S3B), and α-Tubulin as a loading control (T5168), and appropriate HRP-conjugated secondary antibodies. Membranes were stripped of primary and secondary antibodies to allow re-probing using Western Re-Probe (G-Biosciences), according to the manufacturers’ instructions. Labeled antibodies were detected with Amersham ECL Western Blotting Detection Reagents according to the manufacturer’s instructions (GE Healthcare UK Limited, Buckinghamshire, UK), with the results recorded using a cooled charge-coupled device camera system (Fuji-LAS-4000, Fujifilm Life Science Systems, Stamford, CT, USA). Signal intensities of protein bands were quantitated where required using Fujifilm Multigauge software, v3.0 (Stamford, CT, USA).

*Ovary Preparation, Tissue Fixation, Immunofluorescence:* Whole mouse isolated ovaries were placed in Bouin’s fixative for 8–12 h, washed in 70% ethanol, paraffin embedded, and serially sectioned (4 μm thick) throughout the entire ovary, 3 sections per slide, with every 4th slide counterstained with hematoxylin and eosin (H & E). Antibodies specific for; MSI1, MSI2, Caspase-3 (ab13847, Abcam) were used to probe ovary sections using the same protocol: sections were deparaffinized, rehydrated, and permeabilized via heat-mediated antigen retrieval. Sections were then blocked in 3% BSA/PBS for 1.5 h at room temperature, then incubated with primary antibody of choice diluted in 1% BSA/PBS for 15–20 h at 4 °C. After washing in PBS containing 0.05% triton X-100, sections were incubated with the appropriate fluorescent conjugated secondary antibodies (IgG Alexa Fluor Conjugate 633/594/488) diluted in 1% BSA/PBS for 1 h at room temperature. Sections were counter-stained with 4'-6-Diamidino-2-phenylindole (DAPI) and mounted in Mowiol.

*TUNEL*: Sections were treated with 20 μg/mL of proteinase K (Promega, WI, USA) prior to TUNEL analysis using an ApopTag Fluorescein *in situ* Apoptosis Detection Kit (S7110, Merck Millipore, MA, USA) according to the manufacturer’s protocol. Sections were counter-stained with DAPI and mounted in Mowiol.

*Oocyte Collection*: Female mice were super-ovulated via intraperitoneal injection of 10 IU of Folligon (equine chorionic gonadotropin; Intervet, Sydney, Australia) followed by intraperitoneal administration of 10 IU of Chorulon (human chorionic gonadotropin [hCG]; Intervet) 48–50 h later. Cumulus-intact oocytes were recovered 12–15 h after the final hCG injection by rupturing the oviductal ampullae of super-ovulated animals in M2 medium (Sigma-Aldrich). Adherent cumulus cells were then dispersed by treating the collected oocytes with 300 IU/mL hyaluronidase solution. Oocytes were: washed in M2 media and prepared for zona-pellucida binding; fixed in 4% paraformaldehyde/PBS with 0.5% triton X-100 for 30 m or washed in PBS × 3; and collected for RNA or protein extraction.

*Granulosa cell isolation:* 20–40 neonatal mouse ovaries were dissociated in DMEN/F12 HAM with 0.02% Collagenase (Type II) and 0.02% DNAse I (Roche) by incubation at 37 °C in 5% CO2 for 60 min. Ovaries were centrifuged at 2000× *g* for 5 min. The medium was removed and ovaries were resuspended in Trypsin/EDTA in PBS, and incubated at 37 °C in 5% CO2 for 15 min followed by gentle pipetting which completely dissociated cells. FCS was added to inhibit trypsin and cells were centrifuged for 5 min at 2000× *g*. Supernatant was removed and cells were resuspended in a primary ovarian cell culture medium (10% FCS, 0.2% BSA, 2% penicillin-streptomycin, 1% insulin transferrin-selenium, 0.5% 200 mM L-glutamine in DMEM F12 HAM media) to give a final concentration of approximately 5 × 10^5^ cells/mL. If cells were >90% viable (as measured by trypan blue) they were used for primary cell culture. Five hundred microliters of cells were added per well of a 24-well plate (Sarstedt). Ten microliters of 5 mg/mL filtered ascorbic acid were added to each well and cells were left to adhere for 12 h. The medium was removed and replaced with fresh culture medium and cells were cultured for a further 24 h following. Adherent granulosa cells were trypsinized, quenched, and washed 3 × 5 min in PBS before prior to RNA or protein extraction.

*Immunocytochemistry*: Immunocytochemistry was performed on fixed oocytes using primary antibodies for MSI1, MSI2, or anti-α-tubulin (A11126). Oocytes were blocked in 7% goat serum/PBS-0.1% Tween-20 then incubated in primary antibody of choice overnight at 4 °C in 1% BSA in PBS-0.1% Tween-20. Oocytes were washed prior to incubation with the appropriate fluorescent conjugated secondary antibodies (IgG Alexa Fluor Conjugate 633/594/488) diluted in 1% BSA in PBS-0.1% Tween-20 for 1–2 h at room temperature. Oocytes were counter stained in Hoechst (20 μg/mL) and mounted in citifluor.

*Follicle Counts:* All follicles with a visible nucleus in the first section of every H & E stained ovary slide were counted (*i.e.*, ~every 40 μm). Primordial follicles were classified as those with a single layer of squamous granulosa cells. Primary follicles were classified as those that contained cuboidal granulosa cells in a single layer. Secondary follicles were identified as those with two layers of granulosa cells, and pre-antral follicles were classified as those with more than two layers of granulosa cells. Antral follicles exhibited a visible antrum and included pre-ovulatory follicles.

*Sperm-Zona Pellucida Binding:* Freshly collected MII eggs were examined for sperm-zona pellucida binding as previously described [39]. MII oocytes were incubated with capacitated spermatozoa at 2 × 10^5^ sperm/mL in M2 medium for 30 m. Phase microscopy was used to count sperm heads bound to the zona after oocytes were washed out of sperm-containing media.

*Generation of Msi2Gt/Gt Mice, Fertility Trials:* Msi2-deficient mice were gifted by Professor Thomas Graf, group leader of the Center for Genomic Regulation and UPF, Barcelona, Spain generated via retroviral gene-trap insertion, as previously described [20]. Transgenic mice were identified by genomic-PCR analysis of DNA prepared from tail snips obtained at PND20, as previously described [20]. For fertility trials: Msi2^Gt/Gt^ females (6–8 weeks old) were housed with Wt males. Number and size of litters were recorded over a 6-month period.

*Microscopy:* An Axio Imager A1 epifluorescent microscope (Carl Zeiss MicroImaging Inc., NY, USA) was used to visualize sections with pictures taken using an Olympus DP70 microscope camera (Olympus America, PA, USA). Immunocytochemistry imaging was performed on an Olympus FV1000 using a 60×/1.2 NA UPLSAPO oil immersion objective lens.

*Statistics*: Statistical analysis was performed using JMP11 analysis software (SAS, Buckinghamshire, UK). All experiments were biologically replicated independently a minimum of three times, based on power of analysis. The majority of datasets presented a positively or negatively skewed distribution for which non-parametric Wilcoxon/Kruskal–Wallis testing was administered. In figures * denotes statistical significance, specifically: ***: *p* < 0.001; **: *p* < 0.01; *: *p* < 0.05.

## 4. Conclusions

Understanding the mechanisms that control the processes of folliculogenesis and oogenesis is essential to our knowledge of female fertility and reproductive health. Accelerated follicle atresia is a key factor in premature ovarian failure and early onset menopause—this common cause of female infertility is exacerbated by the increasing trend observed among women in the developing world to postpone pregnancy. Consequently this study which determines the impact of post-transcriptional regulation by the Musashi family of RNA binding proteins during mammalian follicle development is of great importance. Herein we have observed that MSI1 and MSI2 are differentially expression in the developing ovary and oocyte. Furthermore, we have established that MSI2 is required for healthy follicle and oocyte development and fertility, indicating a future need to focus on the mRNA targets and regulatory mechanisms utilized by Musashi during folliculogenesis.

## Supplementary Files

Supplementary File 1
