# Supplementary material for: Knockout of RNA Binding Protein MSI2 Impairs Follicle Development in the Mouse Ovary: Characterization of MSI1 and MSI2 during Folliculogenesis
Source: Biomolecules. 2015 Jun 26;5(3):1228–44. doi: 10.3390/biom5031228 (PMC4598749; doi:10.3390/biom5031228)
Supplement: Supplementary File 1 [file biomolecules-05-01228-s001.pdf]

Supplemental Materials

Table S1. Primer sequences for qPCR primers designed for mouse.

Primer	Forward 5'→3'	Reverse 5'→3'
Msi1	GTTACCCAGGGTTCCAAG	GAGAGGGATAGCTGTGAG
Msi2	GAGTTAGATTCCAAGACGATTG	CTGCTCGAAATACTGCTTT
Cyclophilin A	CGTCTCCTTCGAGCTGTTT	ACCCTGGCACATGAATCCT

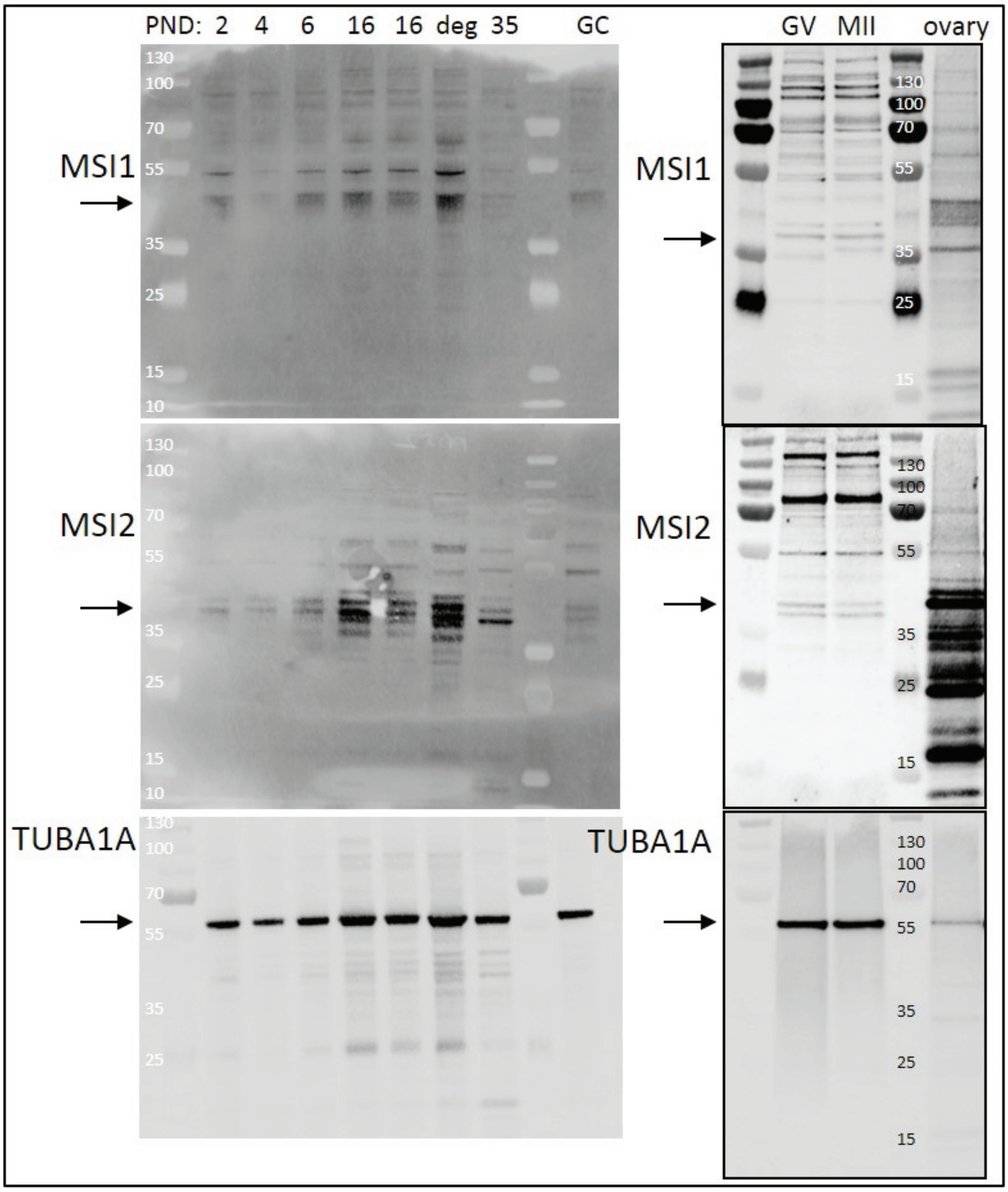

Figure S2. Immunoblot of MSI1 and MSI2 in mouse ovary, granulosa cell and oocyte extracts. **I**mmunoblots of MSI1 and MSI2 in ovary time-course from post-natal day (PND) 2 through to sexual maturity at PND35 and in separate blot GV and MII oocytes, alongside neonate ovary positive control. Positive expression of MSI1 was detected at the appropriate size of 39 kDa for isoform 1 and 34 kDa for isoform 2 (oocytes only). Positive expression of MSI2 was detected at ~37 kDa. Protein expression was measured relative to TUBA1A loading control at 55 kDa. Molecular weight marker S26619 (Thermo scientific). Deg = degraded sample not analysed.

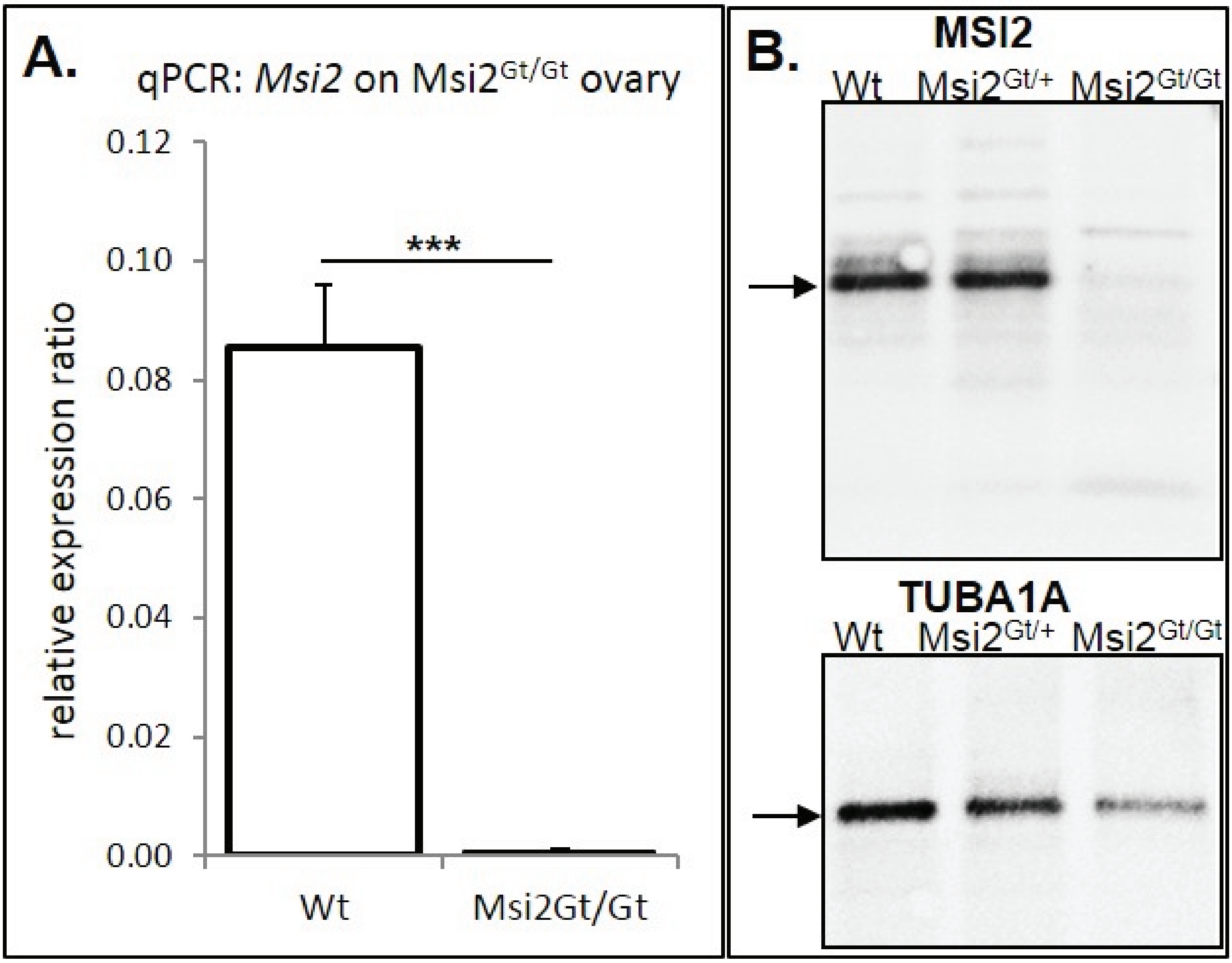

Figure S3. Confirmation of Msi2 knockout in Msi2$^{Gt/Gt}$ adult ovary. (**A**) QPCR analysis of *Msi2* in Msi2$^{Gt/Gt}$ adult (PND35) mouse ovary. MRNA expression relative to *Cyclophilin A* (2eΔCt); values are mean +SEM; N = 3, *** denotes $p < 0.001$. (**B**) Immunoblot analysis of Msi2$^{Gt/Gt}$ adult mouse ovary shows complete loss of full-length MSI2 at ~37 kDa in knockout animals. TUBA1A serves as protein loading control and detects a single band at 55 kDa.

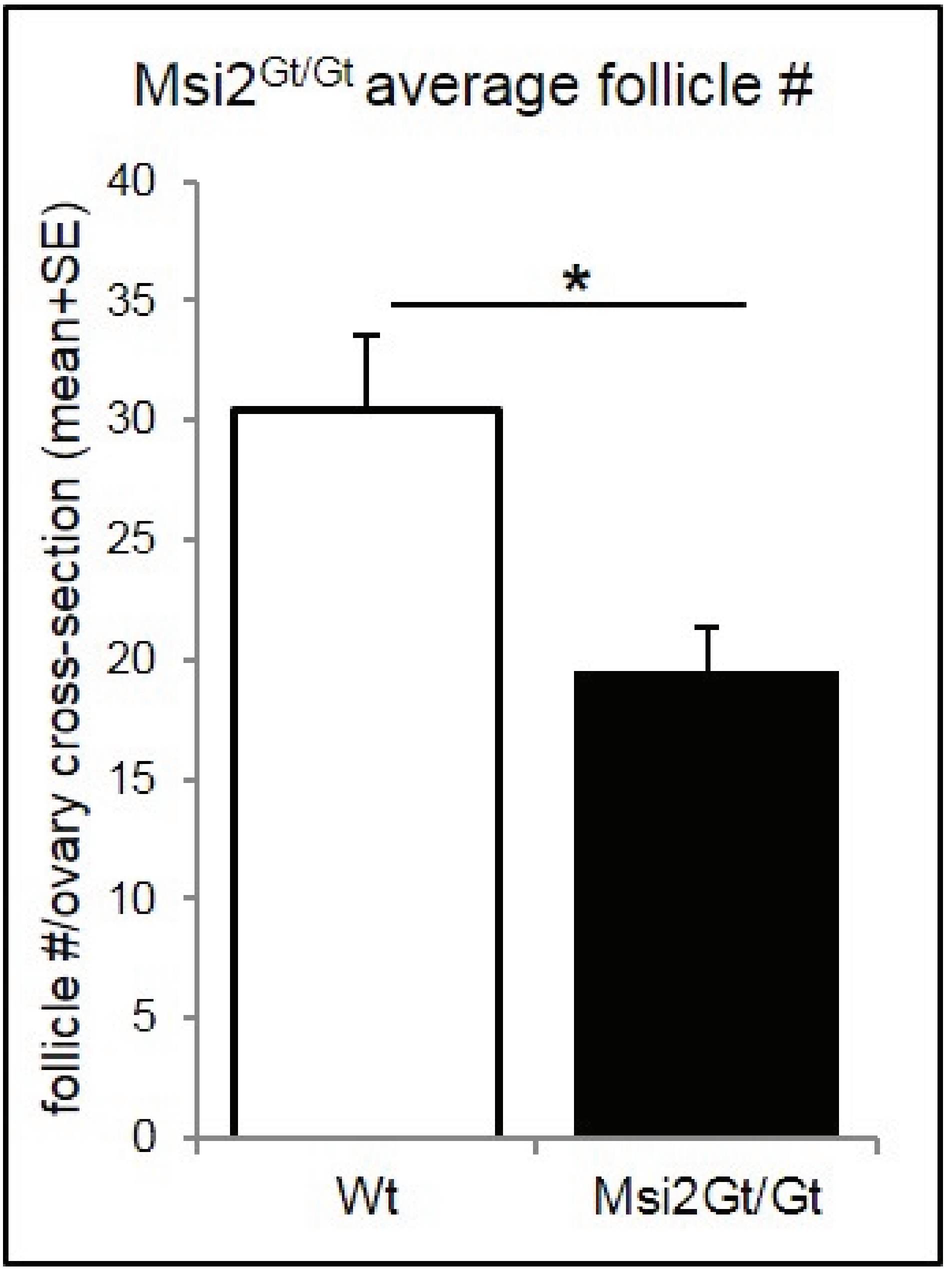

Figure S4. Follicle number in $Msi2^{Gt/Gt}$ adult ovary. Fixed and embedded adult (PND35) $Msi2^{Gt/Gt}$ and Wt ovaries were serially sectioned (4µm thick) throughout the entire ovary, 3 sections per slide, with every 4th slide counterstained with haematoxylin and eosin (H & E). All follicles with a visible nucleus in the first section of every H & E stained ovary slide were counted (*i.e.*, ~ every 40 µm). The data shows average number of follicles per ovary cross-section per ovary +SE; N = 4, * denotes $p < 0.05$.

© 2015 by the authors; licensee MDPI, Basel, Switzerland. This article is an open access article distributed under the terms and conditions of the Creative Commons Attribution license (http://creativecommons.org/licenses/by/4.0/).
